# The effects of low power laser light at 661 nm on wound healing in a scratch assay fibroblast model

**DOI:** 10.1007/s10103-022-03670-5

**Published:** 2022-12-27

**Authors:** Efstathios Giannakopoulos, Annita Katopodi, Michail Rallis, Konstantinos Politopoulos, Eleni Alexandratou

**Affiliations:** 1 Laboratory of Biomedical Optics and Applied Biophysics, School of Electrical and Computer Engineering, National Technical University of Athens, Zografou Campus, 15780 Athens, Greece; 2 Division of Pharmaceutical Technology, School of Pharmacy, National and Kapodistrian University of Athens, Panepistimioupoli, Zografou Campus, 15771 Athens, Greece; 3 Laboratory of Organic Chemistry, School of Chemical Engineering, National Technical University of Athens, Zografou Campus, 15780 Athens, Greece

**Keywords:** Wound healing, Fibroblasts, Scratch, Laser, Red light

## Abstract

Wound treatment, especially for chronic and infected wounds, has been a permanent socio-economical challenge. This study aimed to investigate the ability of red light at 661 nm to accelerate wound healing an *in vitro* wound model using 3T3 fibroblasts. The purpose is further specified in clarifying the mechanisms of wound closure by means of intracellular ROS production, proliferation and migration of cells, and cellular orientation. Illumination effects of red light from a diode laser (661 nm) at different doses on 3T3 cell viability was assessed via MTT assay and tested in a scratch wound model. Wound closure rates were calculated by image analysis at 0, 24, and 48 h after laser treatment. ROS production was monitored and quantified immediately and 24 h after the treatment by fluorescence microscopy. Cellular orientation was quantified by image analysis. No phototoxic energy doses used and increased cell viability in most of the groups. Scratch assay revealed an energy interval of 3 – 4.5 J/cm^2^ that promote higher wound healing rate 24 h post treatment. An increase in ROS production was also observed 24 h post irradiation higher in the group with the highest wound healing rate. Also, cellular orientation toward the margin of the wound was observed and quantified after irradiation. Low power laser light at 661 nm activated both the migration and proliferation in the *in vitro* model used, providing evidence that it could also accelerate wound healing *in vivo*. Also, ROS production and cellular orientation seem to play an important role in wound healing process.

## Introduction

The skin is an organ with a multi-functional role for the organism, such as protecting the human body from external factors and sustaining hydration levels [1]. Hence, the loss of its integrity due to a cutaneous wound could negatively affect the human body. The wound healing process is known for its complexity and the variety of mediators and reactions that take place during four distinct but also overlapping stages: hemostasis, inflammation, proliferation, and remodeling [2, 3]. However, several risk factors (diabetes, infections, smoking) can cause complications and impair wound healing, leading to chronic wounds [4]. Accelerating the wound repair in order to avoid such problems has been a challenge over decades, and several treatments have emerged, for example, wound dressings, tape stripping, or laser irradiation [5].

Wound treatment with irradiation in the red region of the electromagnetic spectrum has drawn the attention of the scientific community since 1993. Since then, numerous studies have been published using irradiation with red light in comparison to other wavelengths in the visible spectrum. Low power laser therapy uses light in the red and mid-IR region of the spectrum to treat wounds in a non-thermal way [6]. The mechanism of action of the red light on wound healing is related to the excitation of skin’s endogenous chromophores, including mitochondrial enzymes, which takes place after the absorption of radiation from the biological tissue [7, 8]. This results in a cascade of events such as reactive oxygen species (ROS) generation, increased ATP synthesis, and calcium oscillations, leading to improved wound healing [9–11].

Several *in vitro* and *in vivo* studies have showed the benefits of red light in the wound healing process [12–15]. However, the heterogeneity of experimental or clinical protocols concerning cells and animals and a variety of experimental irradiation parameters, such as source type, wavelength, fluence, irradiance, pulse duration, repetition regimen, and therapy duration used, have limited the photobiomodulation (PBM) clinical use due to lack of standardized clinical protocols. Another important characteristic of PBM is its biphasic dose response (Arndt–Schulz law). According to this law, very low doses of light have no effect and higher doses have beneficial effect until a plateau is reached. Further increase of the light dose yields in decrease of the beneficial effects until the “no effect” level is reached. Additional increase of the energy dose can be even harmful for the biological system [16].

Fibroblasts are cells that play a pivotal role in wound healing. They are key factors for the proliferative phase since they mediate migration, proliferation, and differentiation of keratinocytes. In addition, fibroblasts from the area surrounding the wound migrate and synthesize collagen, a major event of proliferation stage, which leads to the formation of granulation tissue [17].

The aim of this study was to examine the ability of red light to enhance wound healing in a wound scratch model of 3T3 fibroblasts and to determine the optimum conditions under which this is facilitated. For this purpose, a diode laser (661 nm) was used and different power and energy rates have been tested for their ability to promote fibroblasts proliferation and migration. Furthermore, the generation of ROS was explored with intention to elucidate their role in wound healing process. Image processing and analysis methods have been used to quantify wound closure rate, ROS production, and cellular orientation as a result of PBM.

## Materials and methods

### Cell culture

Μice skin 3T3 fibroblasts were grown in 25 cm^2^ culture flasks in Dulbecco’s modified Eagle’s medium + 4.5 g/L D-glucose, L-glutamine (Gibco), supplemented with 1% antibiotic–antimycotic (Gibco), 0.5% penicillin–streptomycin (Gibco), 0.07% gentamicin solution 1% (Thermo Fisher Scientific), and 10% FBS (Qualified HI/Pen-Str 0.5%, Gibco). Cell cultures were incubated at 37 °C in 5% CO_2_ with 85% humidity. Cells were washed with PBS 1 × (Gibco) and detached with 700 mL/25 cm^2^ Trypsin–EDTA 0.05 (Gibco).

### Irradiation device

Irradiation was performed using a 661-nm diode laser system (FWHM = 5 nm) coupled to an optical fibre and a light diffuser (GCSLS-10-1500 m, China Daheng Group) in order to provide uniform circular illumination spot. At each experiment, irradiation area was centred on the well of interest. Power output at cellular level was assessed using a power meter before and after cellular irradiation. Cell area was homogenous irradiated with the light diffuser with a variability in power less than 2% as measured with the power meter in different points of the irradiated area.

Irradiation parameters, concerning beam and dose aspects, are very important and should be either measured during each experiment at cellular level or appropriately reported to facilitate reproducibility[18].

### Laser irradiation

Cells were seeded in 12-well plates and incubated for 24 h. After the infliction of the scratch, cells were irradiated in 300 μL fresh DMEM with the laser (wavelength 661 nm) from above with the well plate lid off. The different groups were treated with power output density of 5, 10, and 15 mW/cm^2^ for 5, 8, and 13 min, respectively (Table 1). The process was performed in the dark in order to avoid polychromatic light. A well plate of non-irradiated cells, which was treated in the same way, was used as control.

**Table 1:** Irradiation fluence of different groups

	5 min (300 s)	8 min (480 s)	13 min (780 s)
5 mW/cm^2^	1.5 J/cm^2^	2.4 J/cm^2^	3.9 J/cm^2^
10 mW/cm^2^	3 J/cm^2^	4.8 J/cm^2^	7.8 J/cm^2^
15 mW/cm^2^	4.5 J/cm^2^	7.2 J/cm^2^	11.7 J/cm^2^

### Cell viability evaluation and MTT assay

Viability of the cells was assessed by MTT {3-(4,5-dimethylthiazol2-yl)-2,5-diphenyl-2H-tetrazolium bromide, Sigma} assay. 24 h after the irradiation cell medium in each well was replaced with MTT solution (1 mg/mL in DMEM), and cells were incubated for 3 h at 37 °C in 5% CO_2_ with 85% humidity. Then, MTT media was removed and the formazan crystals that had been produced were solubilized with 150 μL DMSO (dimethyl sulfoxide, Sigma). Absorbance was measured at 570 nm using Epoch 2 Microplate Reader (BioTek Instruments). Blank values were measured in wells with DMSO without cells. The relative cell viability was determined as cell survival percentage compared to cells that were treated with complete media, which were used as control. All the experiments were performed five times.

### Scratch assay

The fibroblasts were cultured in 12-well plates (1.5 × 10^5^ cells/well) for 24 h until they form a confluent layer. The scratch was inflicted across the cell layer with a 100-μL sterile pipet tip. The medium was removed, and the cells were washed with PBS in order to remove debris. Next, 300 μL DMEM was added and irradiation was performed. Then, DMEM was added till 1 mL per well and the plates were put back in the incubator. Images were acquired with an inverted light microscope [Olympus ΙX‐81, (Olympus Optical Co., GmbH)] coupled to a CCD camera (XC-30, Olympus} 0, 24, and 48 h post irradiation. All the experiments were performed in triplicate. Image acquisition was performed using the AnalySIS getIT (Olympus Soft Imaging Solutions, GmbH) software. The Image J software was used to measure the area of the scratch. The rate of wound closure was calculated by measuring the area of the scratch, which was not covered with cells 24 and 48 h after irradiation.

### Evaluation of ROS production in cells

The production of ROS in 3T3 cells as a result of the irradiation with red light was examined 0 and 24 h post irradiation. In order to measure ROS production chloromethyl-2′,7′-dichlorodihydrofluorescein diacetate (CM-H2DCFDA, Molecular Probes) was used. The probe was dissolved in EtOH. 10 μg of this solution was diluted with DMEM to obtain 5 μΜ of CM-H2DCFDA. Cells were seeded onto coverslips in culture disks and incubated for 24 h. That solution was added to the cells right after or 24 h post irradiation. Coverslips were incubated in the dark for 30 min. Coverslips with non-irradiated cells were used as control. After the incubation, cells were washed with PBS and the coverslip was placed in a specially designed perfusion chamber allowing live cell imaging [9]. Cells were observed under an epifluorescent upright microscope Olympus BX‐50 (Olympus Optical Co., GmbH) using a 40 × objective lens (UPlanFl, N.A. = 0.75, Olympus) coupled to a CCD camera (XC-30, Olympus). The configuration of the filter cube was U‐MNB excitation BP470‐490, dichroic mirror DM500, and emission BA515. Since CM-H2DCFDA is light sensitive, all the experiments were conducted in the dark.

### Image analysis and ROS quantification

ROS levels were quantified according to the method described in [19]. In brief, images were converted to 8 bit grayscale ones. Five different circular homogenous regions of interest (ROI) were selected from each cell, and the mean intensity of each ROI was calculated with Image J. The same procedure was performed for many cells in each experimental condition in order to provide a sufficient sample from statistical point of view, and the mean value of all the measurements was calculated. The intensity of the images is proportional to the fluorescence intensity of the sample, thus is proportional to the amount of the produced ROS.

### Cellular orientation

Orientation and isotropic properties of cells were characterised using OrientationJ, an ImageJ plug-in [20, 21]. Orientation was visualised and quantified based on the evaluation of the structure tensor in the local neighbourhood for every pixel. Orientation representation, quantitative orientation measurement, and distribution of orientations were provided by the plug-in. Orientation was visualized as colour images with the orientation encoded in a hue-saturation-brightness map where hue is orientation, saturation is coherency, and brightness is the same as the source image. Furthermore, results were used to plot the circular histogram of the normalised distribution of local orientations in every image. The peaks of this histogram point to main cellular orientations along the observed image field.

### Statistical analysis

Data were analysed with the SPSS software (IBM SPSS Statistics, Version 20). Shapiro-Wilks test was used to determine the normality of the data, and one-way analysis of variance (ANOVA) with LSD post hoc test was used to find differences between groups. Statistical significance was considered as *P* < 0.05.

## Results

### Cell viability

**Fig. 1:**
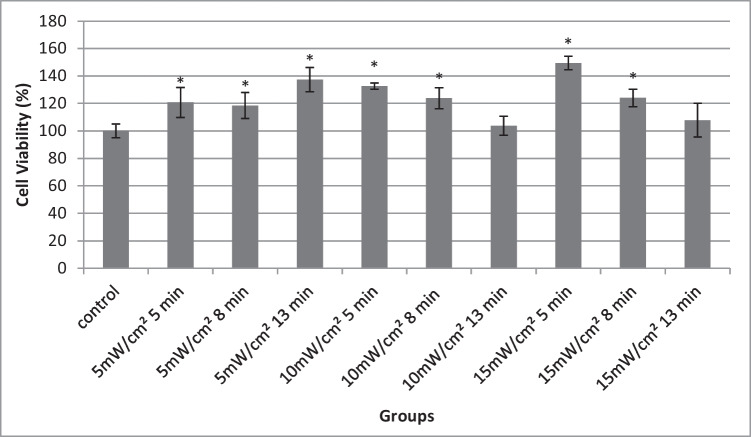
Cell viability results of the different groups 24 h after irradiation on 3T3 fibroblasts. The data are expressed as mean of five experiments. The error bars present standard deviation. *P < 0.05 represents statistically significant differences between experimental and non-irradiated control groups

The effect of red light under different conditions (power output density, irradiation time) on fibroblasts 24 h after the irradiation is shown in Fig. 1. As shown in the figure, neither of the light doses used caused toxicity on the cells. On the contrary in all cases, cell viability was higher than the non-irradiated control. Furthermore, the viability increase was statistically significant in all groups except groups 13 min 10 mW/cm^2^ and 13 min 15 mW/cm^2^, which are the groups where the highest fluence rates were used. Since all the doses used presented no toxic effect, we proceeded in scratch assay experiments for these irradiation conditions.

### Evaluation of treatment with red light of various doses in a scratch wound healing assay

**Fig. 2:**
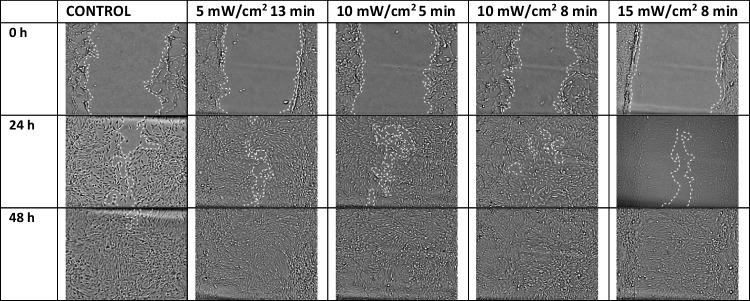
The scratch assay to investigate the wound healing of 3T3 fibroblasts irradiated with different doses of red light (661 nm). Dotted lines indicate the area of the scratch at 0, 24, and 48 h after the treatment. Pictures above present the groups with the better healing rate and the non-irradiated control group

**Fig. 3:**
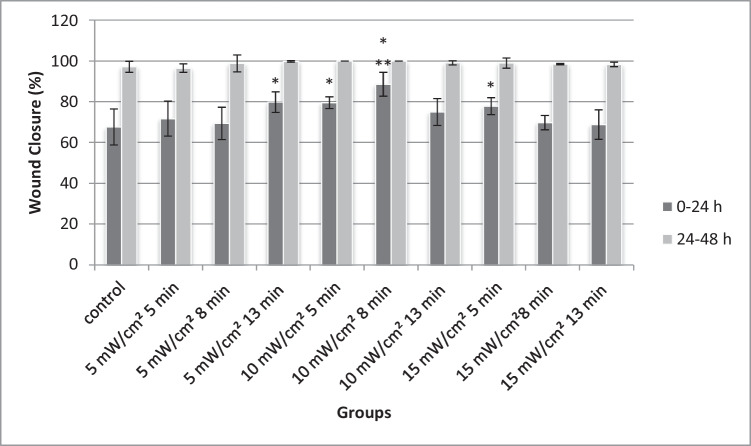
Effect of red light irradiation in 3T3 cells. Scratch wound area was measured 24 and 48 h post treatment. *P < 0.05 represents statistically significant differences between experimental and non-irradiated control groups. **P < 0.05 represents statistically significant differences between 10 mW/cm^2^ for 8 min (4.8 J/cm^2^) group and other irradiated groups

The scratch assay was performed, and images were acquired 0, 24, and 48 h after the treatment (Fig. 2). Irradiated groups were compared with the non-irradiated control group. The effect of red light on wound closure rate can be seen in the graph shown in Fig. 3. As shown in the graph, 24 h after the treatment, doses of 5 mW/cm^2^ for 13 min (3.9 J/cm^2^), 10 mW/cm^2^ for 5 (3 J/cm^2^), and 8 min (4.8 J/cm^2^) and 15 mW/cm^2^ for 5 min (4.5 J/cm^2^) show statistically significant better wound healing rate compared to the control. Furthermore, the group irradiated with 10 mW/cm^2^ for 8 min (4.8 J/cm^2^) shows statistically significant difference of the wound healing rate after 24 h compared to the rest of the groups. Forty-eight hours after irradiation, no significant results were observed as in groups wound closure was almost the same with the control group.

### ROS production in 3T3 cells after the treatment with Red light at 661 nm

**Fig. 4:**
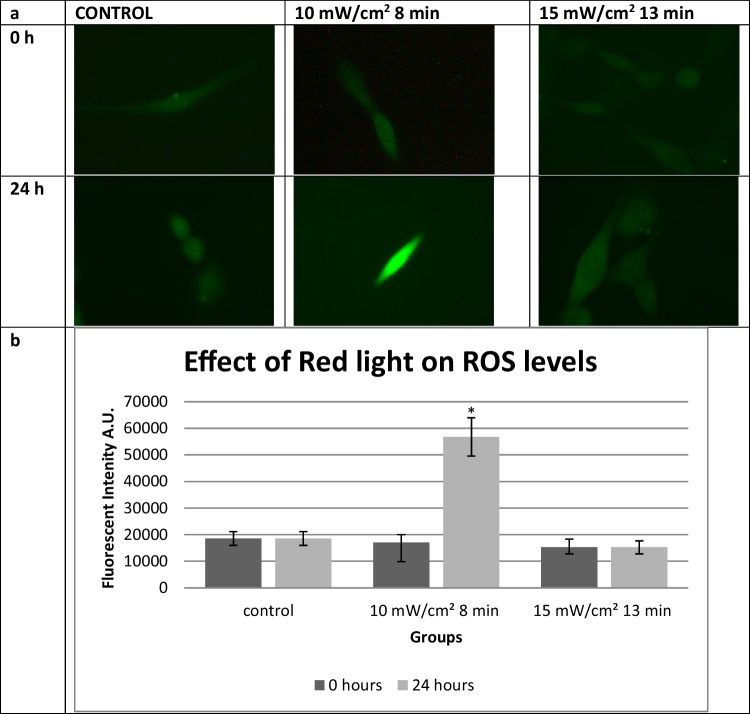
**a** Intracellular ROS levels in 3T3 cells 0 and 24 h after the treatment with red light compared to the non-treated control. **b** Intracellular ROS levels 0 and 24 h after the irradiation with red light at 661 nm. *P < 0.05 represents statistically significant differences between experimental and non-irradiated control groups

In order to investigate ROS production in the irradiated cells, three different groups were selected. The control group, the group that showed the greater wound closing rate, and the one that showed the lesser rate were chosen for further investigation of the formation of intracellular ROS and their contribution to wound closure process. Assessment and quantification of ROS production were examined 0 and 24 h after the treatment. In Fig. 4, intracellular ROS levels are presented in the fluorescent images. Immediately after the treatment, no significant change is noticed in the ROS levels between the control and the irradiated groups. However, 24 h after the irradiation, the amount of intracellular ROS is 3 times higher at group treated the beneficial dose of 10 mW/cm^2^ for 8 min compared to the other groups as revealed by image analysis (Fig. 4).

### Cellular orientation

**Fig. 5:**
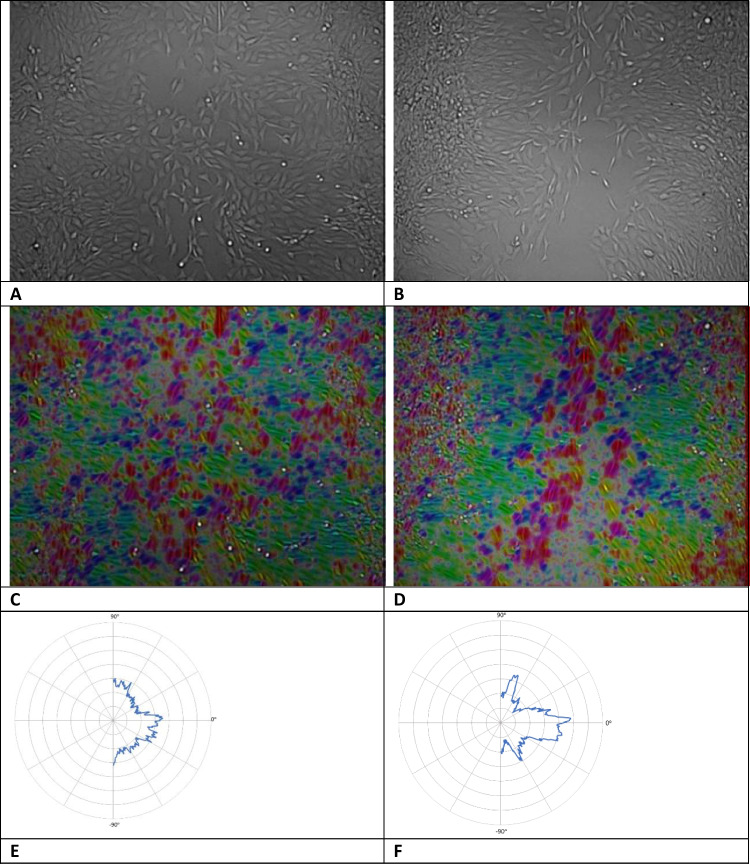
**A**, **B** Initial bright field images of cells. **C**, **D** Hue, saturation, and brightness (HSB) colour maps obtained from the brightfield images after image processing and analysis. These maps assign the same orientation angle to the same colour. **E**, **F** Circular histograms of the normalised distribution of local orientations of the images. On the left column, control cells are shown, while on the right, cells irradiated with 661 nm, at 15 mW and 5 min. Images were acquired 24 h after scratch assay

In the images, 24 h after irradiation with red light, a difference in the direction of the cells migrating to the wound centre was noticed between the control and the groups irradiated with 15 mW/cm^2^. In order to investigate the significance of this hypothesis, the orientation of cells at the area near the wound margin was visualised and quantified, using the ImageJ plug-in OrientationJ. In Fig. 5, brightfield images of control group (A) and irradiated group with 15 mW/cm^2^, 5 min (B) are shown. In the same image (C and D), hue, saturation, and brightness (HSB) colour maps are displayed, obtained from the brightfield images after image processing and analysis with OrientationJ. These maps provide spatial information of cellular orientation as the same orientation angle is assigned to the same colour. In the third row, the corresponding circular histograms of the normalised distribution of local orientations are shown providing quantitative information of the orientation angles. During healing, cells of the control group demonstrate an isotropic distribution of orientations with no favourable orientation, as shown from both colour map (Fig. 5C) and circular histogram of orientations (Fig. 5D). On the other hand, irradiated cells present areas of the same orientations, same colour in the HSB colour map (Fig. 5D). Cells near wound margin appear elongated and oriented towards the wound. The circular histogram of irradiated cells (Fig. 5F) demonstrates that there are 3 peaks that correspond to 3 favourable orientation angles, around (− 80°, − 90°), (80°, 90°) that are the same due to symmetry and 0°. Orientation of 0° corresponds to cells near the wound margin, appears green to HBS maps, while 90° to cells inside the wound, appears red to the colour map. No significant orientation was observed in any other experimental groups at least for the time points that the images were acquired.

## Discussion

Wound healing is known to be a convoluted process, where many factors, that can or cannot be modified, often endanger its course. These risk factors display a great economical problem for physicians that has to be overcome by developing efficient and practical treatment practices [22]. Recently, therapies that utilize light emerging from lasers at the visible area have been developed in order to face the challenge of enhancing wound healing [23]. Specifically, red light has shown potential in accelerating wound healing by promoting proliferation of different cells such as fibroblasts and keratinocytes [24, 25]. These findings suggest a promising approach against delayed wound healing that leads to chronic wounds, since impaired wound healing is connected to dysfunctions in the proliferation of key cells. These events lead to problematic immune function followed by decreased angiogenesis on the wounded area [26].

In the current study, 3T3 mice fibroblasts were used, based on the role of this type of cells on a healthy wound healing. Proliferation and migration of fibroblasts are important for formation of ECM (extracellular matrix) and granulation tissue [27, 28]. The present study is an attempt to optimize laser parameters such as wavelength, power density, and fluence rate that could lead to faster wound closure. Fluence rates from 1.5 to 11.7 J/cm^2^ were tested with different combinations of laser power density and irradiation time. Since neither of the doses tested reduced cell viability, all of them were examined further in a scratch assay model. The scratch assay results demonstrated that doses of 5 mW/cm^2^ for 13 min (3.9 J/cm^2^), 10 mW/cm^2^ for 5 (3 J/cm^2^), and 8 min (4.8 J/cm^2^) and 15 mW/cm^2^ for 5 min (4.5 J/cm^2^) seem to improve wound healing. These suggest that there is a beneficial fluence interval between 3 and 4.8 J/cm^2^ that positively affects fibroblasts in the selected wound model going along with findings of previous works [24, 29]. For fluence rates outside this range, no significant effect was observed. Under the present conditions, no inhibitory effect found and the results seem to be independent of power density values. Among the groups that showed faster wound closure the group of 10 mW/cm^2^ for 8 min (4.8 J/cm^2^) presented statistically significantly higher wound closing rate compared to all other groups, suggesting that fluence around 4.8 J/cm^2^ approaches the optimum dose in the present experimental conditions. These results correspond with those of Ayuk [6] and Mehvahr [15] who used fluence rates of red light between 4.5 and 5 J/cm^2^ in their wound healing experiments.

Based on the results of cell viability MTT tests, the absorbance of all the experimental groups was higher than the control, pointing to higher cell population. This indicates that red light at 661 nm in the specific fluence rate could promote fibroblast proliferation. Interestingly, even though the group with the highest cellular proliferation (15 mW/cm^2^ for 5 min (4.5 J/cm^2^)) accelerated wound closure, it did not present the highest wound closure rate. The group of 4.8 J/cm^2^ fluence rate although it did not present the highest proliferation, it demonstrated the fastest wound healing. Also, there were experimental groups that showed higher proliferation compared to control but did not present faster wound healing. Therefore, it can be hypothesized that another factor possibly fibroblast migration is also engaging with the closure of the scratch wound model. This hypothesis is further supported by the work of Houreld [12] and Sperandio [30] that suggested the role of red light on cell migration. All these findings suggest that the contribution of both migration and proliferation is necessary to promote wound healing.

PBM is believed to enhance wound healing via an increase in ROS production which leads to higher ATP levels [31]. It is well known that high levels of ROS have been linked with a plethora of negative implications on the cell survival and could impair wound healing [32, 33]. However, some studies indicate their beneficial role in the proliferation of cells, when ROS production remains under certain levels [34, 35]. Specifically, when low levels of ROS are produced for a small amount of time, they can act as a trigger and promote proliferation of fibroblasts and the production of collagen and consequently enhance wound healing [36, 37]. In the present study, the intracellular production of ROS was examined and quantified immediately and 24 h after irradiation with red light. Interestingly, the levels of ROS right after the treatment show no difference between the irradiated groups and the control, whilst 1 day after the treatment, the group that was irradiated with power output density of 10 mW/cm^2^ for 8 min showed vastly higher intracellular ROS levels compared to the other groups, suggesting that ROS production is not an abrupt response to irradiation but it starts happening at a time point between 0 and 24 h after the therapy. In one of our previous studies, we have shown that HFFF2 fibroblasts responded to red laser stimulation (1.5 mJ/cm^2^) with an immediate ROS generation. These findings agree with those of Pavlov et al. who found in a mouse PBM model that ROS generation was higher than the control after the 7th day of the treatment [38]. On the other hand, irradiation with red light of 15 mW/cm^2^ for 13 min presented different results. The levels of intracellular ROS seem similar right after the irradiation and 24 h later. A possible explanation could be that between the 24 h after the irradiation, ROS reached threating levels for the cell survival, cellular antioxidant defense mechanisms were activated, and the levels were restored to normal. In addition, the inability of red light at higher fluence rates to accelerate wound healing could be due to the levels of oxidative stress produced. It looks like the higher fluence rates produce a stronger oxidative stress stimulating intracellular antioxidant mechanisms that inhibit the proliferation and migration of the fibroblasts.

Concerning orientation, image processing and analysis provided both spatial and quantitative information of orientation angles. It was noticed that cells of the control group seem to have a rather isotropic distribution with no favourable orientation angle. Interestingly, red light caused a cellular orientation towards the margin of the wound. According to our knowledge, it is the first time that such a cellular response to laser stimulation was reported. In a recent study on wound healing, electric field was used to provide directional signals for the cells to migrate to the wound centre. This directional migration showed that cells had similar shapes and common arrangement, which led to faster wound healing [38]. These results contribute to the hypothesis that causing a directional cell migration could promote wound healing. The ability of red light to stimulate cellular organization and orientation towards wound seems to be favourable of faster wound healing of the irradiated group.

According to our knowledge, it is the first study that uses 3T3 mice fibroblasts in order to shed light on low power red laser light in terms of wound healing process. Another positive aspect of the present study is the report and imaging of ROS generation at the single cell level, providing also the spatial information as a result of photobiomodulation. At last but not least, no cellular orientation after red light low-power irradiation had been reported since now as this information cannot be obtained in cell population studies.

Originally, a large population of cells is the subject of cell analysis and the obtained results are the average of their conduct. Nevertheless, heterogeneity makes its appearance throughout the population and important information related to single individual cells is lost. Additionally, spatial information on biomolecules is also extinct in cell population studies although it has a strong connection to biological processing that actually occurs at the single cell level.

The findings of the current study aimed to contribute to a better understanding of the beneficial role of laser stimulation to wound healing. As other important key players in wound healing are keratinocytes, it would be of special interest to study and reveal their response to red low power laser stimulation. Moreover, further studies should be carried out to translate current findings to *in vivo* experiments in small animals wound models.

## Conclusions

This paper aimed to investigate the ability of low power laser light (661 nm) to accelerate wound healing in a wound scratch model of 3T3 fibroblasts. Image processing and analysis were engaged to quantify the observed changes by means of wound closure rate, ROS production, and cellular orientation. Different laser power and energies were examined in order to optimize laser parameters. The study of cell proliferation and wound closure rate revealed the contribution of both proliferation and migration in wound closure after laser irradiation. No ROS production was observed immediately after laser stimulation, but interestingly, a large amount of ROS was detected 24 h after irradiation in the group that showed the fastest wound close rate in our experimental conditions. Finally, cellular orientation towards the margin of the wound was observed revealing one rather unknown step in the wound healing procedure. As a conclusion, the results of this research gave a better insight into the role of low power red laser on acceleration of wound healing and its underling mechanisms.
